# Meritocracy beliefs and empathic concern among Chinese college students: a moderated serial mediation model of zero-sum thinking and Protestant work ethic

**DOI:** 10.3389/fpsyg.2026.1837514

**Published:** 2026-08-18

**Authors:** Yinfan Zhang

**Affiliations:** Faculty of Applied Foreign Languages, Shantou Polytechnic, Shantou, Guangdong, China

**Keywords:** empathy, intellectual humility, meritocracy belief, Protestant work ethic, social cognitive theory, zero-sum thinking

## Abstract

As meritocratic values have become increasingly emphasized in educational contexts, it is important to understand how students’ endorsement of meritocracy belief is associated with empathic concern. Based on Bandura’s Social Cognitive Theory, this cross-sectional study constructed a moderated serial mediation model to examine the associations between meritocracy belief and empathic concern among Chinese college students, as well as the mediating roles of zero-sum thinking and Protestant work ethic and the moderating role of intellectual humility. Data from 1,986 Chinese college students recruited through Credamo were analyzed using structural equation modeling (SEM). The results indicated that the direct association between meritocracy belief and empathic concern was relatively weak (*β* = −0.067, *p* = 0.034). However, significant indirect associations were observed through zero-sum thinking (indirect effect = −0.079) and Protestant work ethic (indirect effect = −0.105), and through the sequential pathway of zero-sum thinking and Protestant work ethic (indirect effect = −0.029). Furthermore, intellectual humility significantly moderated the association between Protestant work ethic and empathic concern (interaction *β* = 0.170, *p* < 0.001), and this moderating effect extended to the overall chain mediation path. These findings suggest that meritocracy belief is not directly associated with empathic concern to a substantial extent. Rather, its relationship with empathic concern primarily operates through cognitive pathways involving zero-sum thinking and Protestant work ethic, with intellectual humility serving as an important boundary condition. They extend the application of Social Cognitive Theory and provide practical implications for fostering empathic concern among college students.

## 1 Introduction

Meritocracy refers to the principle that social rewards and opportunities should be allocated based on individuals’ ability and effort rather than factors unrelated to personal achievement (Young, 1958; Mijs, 2016). In educational contexts, meritocratic values emphasize that academic success is primarily determined by students’ ability and effort and are reflected through achievement-based evaluation and selection practices (Wiederkehr et al., 2015; Korthals and Dronkers, 2016; Johnston et al., 2024). Through continuous exposure to such contexts, students may gradually internalize these values and develop individual-level meritocracy beliefs, referring to the extent to which they endorse the idea that effort and ability determine academic success (Batruch et al., 2023). As a belief system concerning the relationship between effort, ability, and achievement, meritocracy belief may shape how individuals interpret success, failure, and personal responsibility. These interpretations may further influence how individuals perceive others and respond in interpersonal situations. Empathic concern, as an affective component of empathy, reflects individuals’ sympathy and concern for others and plays an important role in interpersonal relationships and psychological well-being among college students (Jones and Doolittle, 2017; Mella et al., 2021; Kim et al., 2024). Therefore, examining the relationship between meritocracy belief and empathic concern may provide insight into the broader interpersonal implications of meritocratic beliefs.

Existing research on meritocracy belief has primarily focused on its associations with academic achievement, perceptions of social mobility, and the justification of social inequality (Wiederkehr et al., 2015; Mijs, 2021; Olivos, 2021; Blouin et al., 2025; Matamoros-lima et al., 2025). These studies have provided valuable insights into how meritocracy belief shapes individuals’ achievement-related cognition and social perceptions. However, relatively little attention has been paid to its potential association with empathic concern and the cognitive processes underlying this relationship. From the perspective of Social Cognitive Theory, individuals’ beliefs influence their emotional responses through cognitive interpretations of social situations (Bandura, 1986). Accordingly, meritocracy belief may be associated with empathic concern through specific cognitive orientations. Zero-sum thinking represents one possible pathway because it emphasizes competition, resource scarcity, and the perception that one individual’s success may occur at the expense of others (Meegan, 2010; Davidai, 2025). Protestant work ethic represents another possible pathway because it emphasizes the moral value of effort and attributes success or failure primarily to individual responsibility (Bernardo et al., 2018). Although Protestant work ethic originated from Western cultural traditions, its emphasis on diligence, perseverance, and effort-based achievement also resonates with Confucian values in Chinese culture, which similarly emphasize hard work, persistence, and respect for educational achievement (Zhang et al., 2012). Therefore, examining Protestant work ethic among Chinese college students may help clarify its association with empathic concern in a non-Western educational context. Together, these two cognitive orientations may provide insight into how meritocracy belief relates to empathic concern. However, previous studies have largely examined these constructs separately (Rosenthal et al., 2011; Zúñiga et al., 2022; Davidai and Tepper, 2023; Wang and Ying, 2026), leaving their potential sequential relationship insufficiently explored.

To address these research gaps, the present study examines whether individual differences in meritocracy belief are associated with college students’ empathic concern, whether zero-sum thinking and Protestant work ethic sequentially account for this association, and whether intellectual humility moderates the association between Protestant work ethic and empathic concern. Guided by Bandura’s (1986) Social Cognitive Theory, this study constructs a moderated serial mediation model to examine the cognitive pathways associated with meritocracy belief and empathic concern.

## 2 Literature review

### 2.1 Theoretical framework

Bandura’s Social Cognitive Theory (SCT) provides a theoretical framework for understanding how individuals’ cognitive beliefs are formed within social contexts and how these beliefs are associated with affective and behavioral outcomes (Bandura, 1986, 2001). Centered on the triadic reciprocal determinism, this theory explicitly states that dynamic interactions exist among individual behavior, internal cognitive systems, and the external environment (Bandura, 1986). From this perspective, individuals are not passive recipients of environmental influences. Instead, they actively interpret social experiences through cognitive processes, gradually developing relatively stable beliefs and behavioral tendencies. Through mechanisms such as observational learning, self-regulation, and self-evaluation, individuals transform contextual information into personal cognitive orientations that guide subsequent emotional and behavioral responses (Schunk and Usher, 2012; Zimmerman, 2000). Therefore, SCT offers a useful framework for examining how socially shaped beliefs are associated with individuals’ social–emotional responses in educational settings (Schunk and DiBenedetto, 2023).

Within this framework, meritocracy belief in the present study is conceptualized as an individual-level cognitive belief rather than an objective characteristic of the educational environment. Although meritocratic values may exist within broader social and educational contexts, the present study focuses on individuals’ internalization and endorsement of these values. From the perspective of SCT, meritocracy belief may shape how individuals perceive achievement, responsibility, and interpersonal situations. Individuals who strongly endorse meritocracy beliefs may be more likely to interpret achievement through competitive and effort-centered perspectives, which may strengthen zero-sum thinking by emphasizing limited opportunities and competition among individuals, and reinforce Protestant work ethic by attributing academic success primarily to personal effort and ability. These cognitive orientations may further be associated with empathic concern by shaping how individuals evaluate others’ circumstances.

Meanwhile, SCT highlights that individual characteristics may shape the extent to which cognitive beliefs are associated with affective and social responses (Bandura, 2001). Individuals with different dispositions may interpret and respond to similar cognitive orientations in different ways, suggesting that personal traits can serve as important boundary conditions in the relationship between cognitive orientations and socio-emotional responses. Related research has also suggested that individual differences influence how cognitive factors are linked to affective outcomes, highlighting the importance of considering personal characteristics within the SCT framework (De la Fuente et al., 2022). In this study, intellectual humility is considered a dispositional characteristic that may represent such a boundary condition, reflecting individual differences in how cognitive orientations are associated with empathic concern.

Based on the above theoretical reasoning, this study proposes an SCT-based framework to explain how meritocracy beliefs are associated with college students’ empathic concern through cognitive orientations. Meritocracy belief is viewed as an individual-level value belief that may influence how students interpret success and failure. Zero-sum thinking and Protestant work ethic are considered cognitive orientations that reflect different ways of understanding competition, responsibility, and effort, whereas empathic concern represents a social–emotional outcome. Intellectual humility is further included as an individual characteristic to examine whether personal differences influence the association between cognitive orientations and empathic concern.

### 2.2 Definition of core variables

#### 2.2.1 Meritocracy belief (MB)

Meritocracy belief refers to the extent to which individuals endorse the idea that success is primarily determined by personal ability and effort (Young, 2017). In educational contexts, meritocracy belief is mainly reflected in the high emphasis on academic ranking, admission opportunities, and competitive evaluation systems (Rafalow and Puckett, 2022). Within the framework of SCT, meritocracy belief can be understood as a cognitive belief formed through individuals’ interpretation and internalization of contextual values.

#### 2.2.2 Empathic concern (EC)

Empathic concern refers to an affective tendency characterized by sympathy and concern for others who are experiencing difficulties (Davis, 1980). It reflects individuals’ emotional responses toward others, including feelings of compassion, concern, and sympathy in response to others’ situations (Cuff et al., 2016). Previous research has shown that empathic concern is associated with prosocial behaviors and interpersonal functioning among students (Smits et al., 2011). In the present study, empathic concern is conceptualized as an affective outcome reflecting students’ emotional responses toward others.

#### 2.2.3 Zero-sum thinking (ZST)

Zero-sum thinking refers to a cognitive orientation in which individuals perceive social resources as limited and fixed and believe that one person’s gains necessarily imply another person’s losses (Różycka-Tran et al., 2015). In educational contexts characterized by competition and limited opportunities, zero-sum thinking may lead students to perceive peers’ success as potentially conflicting with their own interests, thereby viewing academic achievement as a competitive rather than mutually beneficial process (Labaree, 2011). From the perspective of SCT, zero-sum thinking can be understood as a cognitive orientation that may develop through individuals’ interpretation of social contexts characterized by competition.

#### 2.2.4 Protestant work ethic (PWE)

Protestant work ethic refers to a belief system that assigns moral value to hard work and regards diligence as an important basis for personal worth, while viewing laziness as a negative personal characteristic (Weber and Kalberg, 2013; Furnham, 2021). In educational contexts, this value orientation is reflected in individuals’ endorsement of effort as an important pathway to academic achievement. Although Protestant work ethic shares an emphasis on effort with meritocracy belief, the two constructs represent different psychological orientations. Meritocracy belief concerns beliefs about the determinants of success (Batruch et al., 2023), whereas Protestant work ethic emphasizes the moral value of effort itself. From the perspective of SCT, Protestant work ethic can be understood as a cognitive orientation associated with individuals’ interpretation of achievement and responsibility in social contexts.

#### 2.2.5 Intellectual humility (IH)

Intellectual humility reflects a personality trait characterized by a clear awareness of one’s own cognitive limitations, an open attitude toward diverse perspectives, and avoidance of absolutizing one’s own judgments (Gregg and Mahadevan, 2014; McElroy et al., 2014; Leary et al., 2017). In educational and social comparison contexts, individuals with high intellectual humility are more likely to recognize their own cognitive limitations, respect individual differences, and accept diverse viewpoints with an open attitude, thereby reducing defensive and confrontational cognitive tendencies (Porter and Schumann, 2018; Wong and Wong, 2021). From the perspective of SCT, intellectual humility can be considered an individual characteristic that may shape how cognitive beliefs are associated with affective outcomes.

### 2.3 Hypothesis development

Based on Bandura’s Social Cognitive Theory (SCT), individuals’ affective and behavioral outcomes are shaped through dynamic interactions among contextual influences, cognitive processes, and personal characteristics (Bandura, 1986, 2001). In educational contexts, meritocratic values emphasizing effort, ability, and achievement may be internalized by students and reflected in individual-level meritocracy beliefs. These beliefs may further relate to students’ interpretations of social relationships, achievement attribution, and emotional responses toward others. On the basis of the above theoretical framework and existing research findings, this study put forward the following research hypotheses regarding the mechanism through which meritocracy belief affects students’ empathic concern.

Meritocracy belief emphasizes the idea that success is primarily determined by personal ability and effort. From the perspective of SCT, individual cognitive beliefs provide interpretive frameworks for understanding social information and evaluating others’ situations (Bandura, 1986). Students who strongly endorse meritocracy beliefs may be more likely to interpret others’ achievements and difficulties by focusing on effort and ability, paying less attention to situational influences, which may weaken empathic concern (Kosse et al., 2026). Moreover, the emphasis on personal achievement in meritocracy belief may encourage students to focus more on their own goals and performance evaluation (Dalla-Zuanna et al., 2025), thereby reducing attention to others’ experiences and emotional resonance (Feng et al., 2013). Based on this, this study proposes Hypothesis H1: Meritocracy belief is negatively associated with students’ empathic concern.

According to SCT, individuals’ cognitive beliefs influence how they process social information and interpret social relationships (Bandura, 1986). Students who strongly endorse meritocracy beliefs may be more likely to interpret achievement outcomes through a competitive framework, perceiving success and opportunities as limited resources distributed among individuals. Such perceptions may strengthen their sense of resource scarcity and be associated with stronger zero-sum thinking (Sun and Xiong, 2023; Davidai and Tepper, 2023). Accordingly, this study proposes Hypothesis H2: Meritocracy belief positively predicts zero-sum thinking.

Zero-sum thinking is a typical competitive cognitive bias. Existing empirical studies have confirmed that this cognitive tendency significantly weakens individuals’ expression of empathic concern (Wang and Ying, 2026). In competitive contexts, individuals’ empathic concern toward competitors are markedly inhibited, and emotional resonance with others’ hardships may even be replaced by negative emotions such as schadenfreude (Cikara et al., 2011). Accordingly, this study proposes Hypothesis H3: Zero-sum thinking negatively predicts empathic concern levels.

From the perspective of attribution theory, individuals’ explanations for success and failure shape their subsequent judgments and responses (Weiner, 1985). Individuals who strongly endorse meritocracy beliefs tend to view achievement as closely related to personal effort and ability, which may strengthen their tendency to attribute outcomes to internal and controllable factors. When effort becomes a primary explanation for achievement, individuals may be more likely to regard hard work as an important and morally meaningful quality, thereby strengthening their Protestant work ethic orientation (Mudrack, 1997; Weber and Kalberg, 2013). Accordingly, this study proposes Hypothesis H4: Meritocracy belief positively predicts Protestant work ethic.

When the Protestant work ethic becomes a stable cognitive belief, this effort-centered attributional framework may extend to judgments of others’ situations. According to the perspective of responsibility judgment in attribution theory, when others’ failure is attributed to internal and controllable factors, individuals are more likely to regard it as a result for which the other party should take responsibility, thereby reducing sympathy and increasing negative emotional responses such as blame (Weiner, 1985, 1995). Accordingly, this study proposes Hypothesis H5: Protestant work ethic negatively predicts empathic concern levels.

Zero-sum thinking reflects the perception of resource scarcity and a win-lose structure, viewing achievement as a limited outcome obtained through competition (Yoo and Pensini, 2025; Davidai and Tepper, 2023). In this situation, individuals are more inclined to seek legitimate explanations for differences in success and failure among individuals (Skitka and Tetlock, 1992). Effort is more likely to be viewed as a legitimate basis for explaining achievement differences (Weiner, 1995), and may therefore become associated with perceived deservingness and moral evaluation (Fwu et al., 2014; Chen et al., 2024). Therefore, zero-sum thinking may strengthen the belief that effort carries moral value and further reinforce Protestant work ethic orientation. Accordingly, this study proposes Hypothesis H6: Zero-sum thinking positively predicts Protestant work ethic.

The SCT suggests that individual traits influence the process of transforming cognitive beliefs into emotional outcomes (Bandura, 1986). Intellectual humility, manifested as recognition of one’s cognitive limitations and openness to different viewpoints (Krumrei-Mancuso et al., 2020), may encourage more flexible judgments and reduce reliance on absolute evaluations (Leary et al., 2017). Given that the Protestant work ethic reinforces the sense of responsibility for personal effort and may be associated with lower empathic concern toward others’ hardships (Christopher and Schlenker, 2005), individuals with high intellectual humility are more likely to maintain openness in judgment when evaluating others’ situations, thus weakening the negative association between Protestant work ethic and empathic concern (Stroud and Murray, 2025). Accordingly, this study proposes Hypothesis H7: Intellectual humility moderates the association between Protestant work ethic and empathic concern, such that the negative association is weaker among individuals with higher levels of intellectual humility.

## 3 Methods

### 3.1 Research design

This study adopted a cross-sectional design to examine the associations between meritocracy belief and empathic concern, including the independent and serial mediating roles of zero-sum thinking and Protestant work ethic, as well as the moderating role of intellectual humility.

### 3.2 Participants

Participants were Chinese college students aged 18 years or older, including undergraduates, master’s students, and doctoral students, recruited through Credamo, a professional Chinese online research platform. Eligible participants were algorithmically matched and invited from Credamo’s participant pool according to the predefined inclusion criteria (i.e., Chinese college students aged 18 years or older). After reading the informed consent statement, participants voluntarily completed the anonymous online questionnaire. A total of 2000 questionnaires were collected. After excluding 14 invalid questionnaires due to routine responses, inconsistent logical answers, or missing items, 1986 valid questionnaires were retained, with a valid response rate of 99.3%. The demographic characteristics of the participants are presented in Table 1. In terms of gender, 1,105 were male (55.6%) and 881 were female (44.4%). For age, 853 were aged 18–20 (43.0%), 637 aged 21–22 (32.1%), 330 aged 23–26 (16.6%), and 166 aged 27 or above (8.3%). Regarding educational level, 1,514 were undergraduates (76.2%), 394 were master’s students (19.9%), and 78 were doctoral students or above (3.9%). The final sample size exceeded the minimum recommendations for structural equation modeling and was considered adequate to ensure stable parameter estimation and sufficient statistical power for testing the proposed model (Kline, 2023; Wolf et al., 2013).

**Table 1:** Descriptive statistics of demographic information.

Item	Selection	Frequency	Percentage (%)
Gender	Male	1,105	55.6
	Female	881	44.4
Age	18–20 years old	853	43
	21–22 years old	637	32.1
	23–26 years old	330	16.6
	27 years old and above	166	8.3
Educational Level	Undergraduate	1,514	76.2
	Master’s student	394	19.9
	Doctoral or above	78	3.9

### 3.3 Ethical considerations

This study was conducted in strict accordance with scientific research ethics and the principles of the Declaration of Helsinki. The research protocol was approved by the Ethics Committee of Shantou Polytechnic. Before questionnaire distribution, participants were fully informed of the purpose and content of the study, as well as the principles of data anonymization and confidentiality. All participants provided voluntary written informed consent and were free to withdraw at any time without penalty. All data were collected and coded anonymously, used solely for academic analysis in this study, kept strictly confidential, and not used for any non-research purposes.

### 3.4 Measurements

All scales were administered in Chinese and rated on a five-point Likert scale (1 = strongly disagree, 5 = strongly agree). Cronbach’s *α* was used to assess the internal consistency reliability of each scale. Since the original instruments were developed in English, all scales were translated into Chinese following established cross-cultural adaptation procedures. Two bilingual researchers independently translated the original English versions into Chinese. The translated versions were then back-translated into English by another bilingual researcher who was blinded to the original instruments. Any discrepancies between the original and back-translated versions were discussed by the research team until consensus was reached to ensure conceptual and linguistic equivalence.

#### 3.4.1 Meritocracy belief

Meritocracy belief was measured using the Meritocracy Scale (Zimmerman and Reyna, 2013), which assesses endorsement of the view that “effort should lead to success.” Four reverse-scored items (“In society, working hard does not automatically lead to success”; “Even if people work very hard, they may not achieve success”; “Discrimination limits some people’s ability to succeed”; “People often succeed because they know the ‘right people’, not because they work hard”) were recoded before data analysis. Higher scores indicate stronger endorsement of meritocracy belief. In this study, Cronbach’s *α* for this scale was 0.888.

#### 3.4.2 Empathic concern

Empathic concern was measured using the Empathic Concern subscale of the Interpersonal Reactivity Index (Davis, 1980), which assesses other-oriented sympathy and concern for those in need. Higher scores indicate higher empathetic concern. Three reverse-scored items (“When someone is having a hard time, I sometimes do not feel very sympathetic”; “In emergencies, I usually do not feel very involved”; “When I see someone being treated unfairly, I sometimes do not feel very much pity for them”) were recoded before data analysis. In this study, Cronbach’s α for this subscale was 0.864.

#### 3.4.3 Zero-sum thinking

Zero-sum thinking was measured using the Zero-Sum Thinking Scale (Różycka-Tran et al., 2015), which assesses the tendency to view social relations and success as inherently competitive and zero-sum. Higher scores indicate stronger zero-sum thinking. In this study, Cronbach’s *α* for this scale was 0.861.

#### 3.4.4 Protestant work ethic

Protestant work ethic was measured using the Protestant Work Ethic Scale (Mirels and Garrett, 1971), which assesses endorsement of the moral value of hard work and the belief that success or failure stems from individual effort. Higher scores indicate stronger adherence to the Protestant work ethic. Based on confirmatory factor analysis (CFA), seven items with standardized factor loadings below 0.50 were removed (ranging from 0.086 to 0.289), including “Most people spend too much time on recreational activities of no real value,” “Money obtained easily, such as from gambling or speculation, is usually spent unwisely,” “The most difficult college courses are usually the most rewarding,” “People should have more time for relaxation and leisure,” “Working hard does not guarantee success,” “Credit cards are like a ticket to irresponsible spending,” and “Life would be more meaningful if we had more leisure time.” These item deletions were based on empirical evidence from CFA, while the retained items continued to capture the central aspects of Protestant work ethic, particularly beliefs regarding diligence, effort, and achievement through hard work. The final 12-item scale demonstrated good internal consistency (Cronbach’s α = 0.929).

#### 3.4.5 Intellectual humility

Intellectual humility was measured using the Intellectual Humility Scale (Leary et al., 2017), which assesses recognition of one’s own cognitive limitations, willingness to admit possible errors, and respect for others’ viewpoints. Higher scores indicate higher intellectual humility. In this study, Cronbach’s α for this scale was 0.863.

### 3.5 Data analysis strategy

Data analysis was performed using SPSS 27.0 and R Studio (version 4.4.0). Data cleaning, descriptive statistics, correlation analysis, and reliability analysis were conducted in SPSS. No missing values were identified after data screening. Confirmatory factor analysis (CFA) and structural equation modeling (SEM) were conducted using the lavaan package in R. The hypothesized model was estimated at the latent variable level using the maximum likelihood (ML) estimator, with the five-point Likert-type items treated as continuous indicators. The final SEM model included five latent constructs measured by 39 observed indicators, along with a latent interaction construct between Protestant work ethic and intellectual humility represented by eight product indicators generated using the double-mean-centering product-indicator approach. Thus, the model included 47 observed indicators in total. The degrees of freedom were calculated as the difference between the number of unique elements in the observed covariance matrix (47 × 48/2 = 1,128) and the number of freely estimated parameters (106), resulting in 1022 degrees of freedom. Bootstrap resampling (5,000 samples) was used to estimate indirect effects and their bias-corrected 95% confidence intervals (CIs). Indirect effects were considered statistically significant when the 95% CI did not include zero. The interaction effect was further examined through simple slope analyses and conditional indirect effect analyses. All statistical tests were two-tailed, with a significance level set at *p* < 0.05.

## 4 Results

### 4.1 Validity analysis

Before testing the structural model, confirmatory factor analysis (CFA) was conducted on the measurement model. The KMO measure of sampling adequacy was 0.956, and Bartlett’s test of sphericity was significant (*χ*^2^ = 38508.612, df = 741, *p* < 0.001), indicating that the data were suitable for factor analysis. The five-factor measurement model consisting of meritocracy belief, empathic concern, zero-sum thinking, Protestant work ethic, and intellectual humility showed satisfactory fit: *χ*^2^/df = 3.62, CFI = 0.952, TLI = 0.949, RMSEA = 0.036, SRMR = 0.043. The standardized factor loadings were acceptable across all constructs, supporting the factorial validity of the measurement model. Convergent validity and composite reliability (CR) were further examined. The CR of each latent variable was above 0.70 (MB = 0.888, EC = 0.866, ZST = 0.862, PWE = 0.929, IH = 0.865), indicating good internal consistency. The average variance extracted (AVE) of all variables exceeded 0.50 except for zero-sum thinking, whose AVE was 0.442, slightly below the 0.50 threshold (MB = 0.534, EC = 0.520, PWE = 0.527, IH = 0.520). Although the AVE of zero-sum thinking was slightly below the recommended criterion, this construct demonstrated satisfactory composite reliability (CR = 0.862), with most standardized factor loadings ranging from 0.587 to 0.796. Therefore, the convergent validity of zero-sum thinking was considered acceptable. Discriminant validity was assessed using the heterotrait–monotrait ratio (HTMT). The HTMT values among all constructs ranged from 0.063 to 0.532, below the recommended threshold of 0.85, indicating satisfactory discriminant validity. Notably, the HTMT value between meritocracy belief and Protestant work ethic was 0.532, suggesting that these two theoretically related constructs were empirically distinguishable. Overall, the measurement model in this study was suitable for subsequent structural model analysis.

### 4.2 Common method bias test

Given that all the data in this study were derived from self-reported questionnaires at the same time point, there may be a potential risk of common methodological bias. Therefore, Harman’s single-factor test and unmeasured latent method construct (ULMC) approach were used for statistical control and testing. Harman’s single-factor test was performed by conducting an unrotated exploratory factor analysis on all items. The results revealed five common factors with eigenvalues greater than 1. The first unrotated factor had an eigenvalue of 10.451 and accounted for 26.797% of the total variance, below the 40% threshold. The five factors together explained 58.018% of the total variance, indicating no single factor dominated most of the variance. The ULMC approach was further adopted by adding a latent method factor to the original five-factor CFA model, so that all items loaded on both their theoretical factors and the method factor with constrained loadings to examine the influence of method factors on model fit. The results showed negligible changes in model fit indices after adding the method factor (ΔCFI = 0.016, ΔRMSEA = 0.006), suggesting that the method factor did not significantly improve model explanatory power. Overall results indicated no severe common method bias in this study, and method factors did not pose a substantial threat to the main conclusions.

### 4.3 Descriptive statistics and correlation analysis

Means, standard deviations, and correlation coefficients for all study variables are presented in Table 2. Descriptive statistics showed that scores for meritocracy belief (*M* = 3.038, SD = 0.668), empathic concern (*M* = 2.960, SD = 0.612), zero-sum thinking (*M* = 3.124, SD = 0.533), and Protestant work ethic (*M* = 2.990, SD = 0.633) were at a moderate level, while the score for intellectual humility (*M* = 2.485, SD = 0.621) was relatively low. Correlation analyses indicated that all pairwise correlation coefficients were significant at the *p* < 0.001 level, and the directions of correlations were consistent with theoretical expectations. Specifically, meritocracy belief was significantly negatively correlated with empathic concern (*r* = −0.200), and significantly positively correlated with zero-sum thinking (*r* = 0.383), Protestant work ethic (*r* = 0.497), and intellectual humility (*r* = 0.169). Zero-sum thinking was significantly negatively correlated with empathic concern (*r* = −0.223) and significantly positively correlated with Protestant work ethic (*r* = 0.403). Protestant work ethic was significantly negatively correlated with empathic concern (*r* = −0.276). Intellectual humility was significantly positively correlated with empathic concern (*r* = 0.085), as well as with zero-sum thinking (*r* = 0.159) and Protestant work ethic (*r* = 0.189). These correlations provided preliminary empirical support for the subsequent tests of the structural model.

**Table 2:** Correlation analysis.

Variable	Mean	SD	MB	EC	ZST	PWE	IH
MB	3.038	0.668	1				
EC	2.960	0.612	−0.200***	1			
ZST	3.124	0.533	0.383***	−0.223***	1		
PWE	2.990	0.633	0.497***	−0.276***	0.403***	1	
IH	2.485	0.621	0.169***	0.085***	0.159***	0.189***	1

MB, meritocracy belief; EC, empathic concern; ZST, zero-sum thinking; PWE, protestant work ethic; IH, intellectual humility. *p < 0.05, **p < 0.01, ***p < 0.001.

### 4.4 Structural model testing for simple mediation, chain mediation, and moderation effects

The hypothesized structural model demonstrated satisfactory fit with the data (*χ*^2^ = 4023.168, df = 1,022, *χ*^2^/df = 3.93, CFI = 0.929, TLI = 0.925, RMSEA = 0.038, 90% CI [0.037, 0.040], SRMR = 0.045). Given the large sample size in this study (*N* = 1986), the chi-square value is prone to significance due to sample size sensitivity. Therefore, CFI, TLI, RMSEA, and SRMR were used as core fit indices. All indices met acceptable criteria, indicating that the structural model fitted the data well and was suitable for subsequent path coefficient and effect analysis.

**Figure 1:**
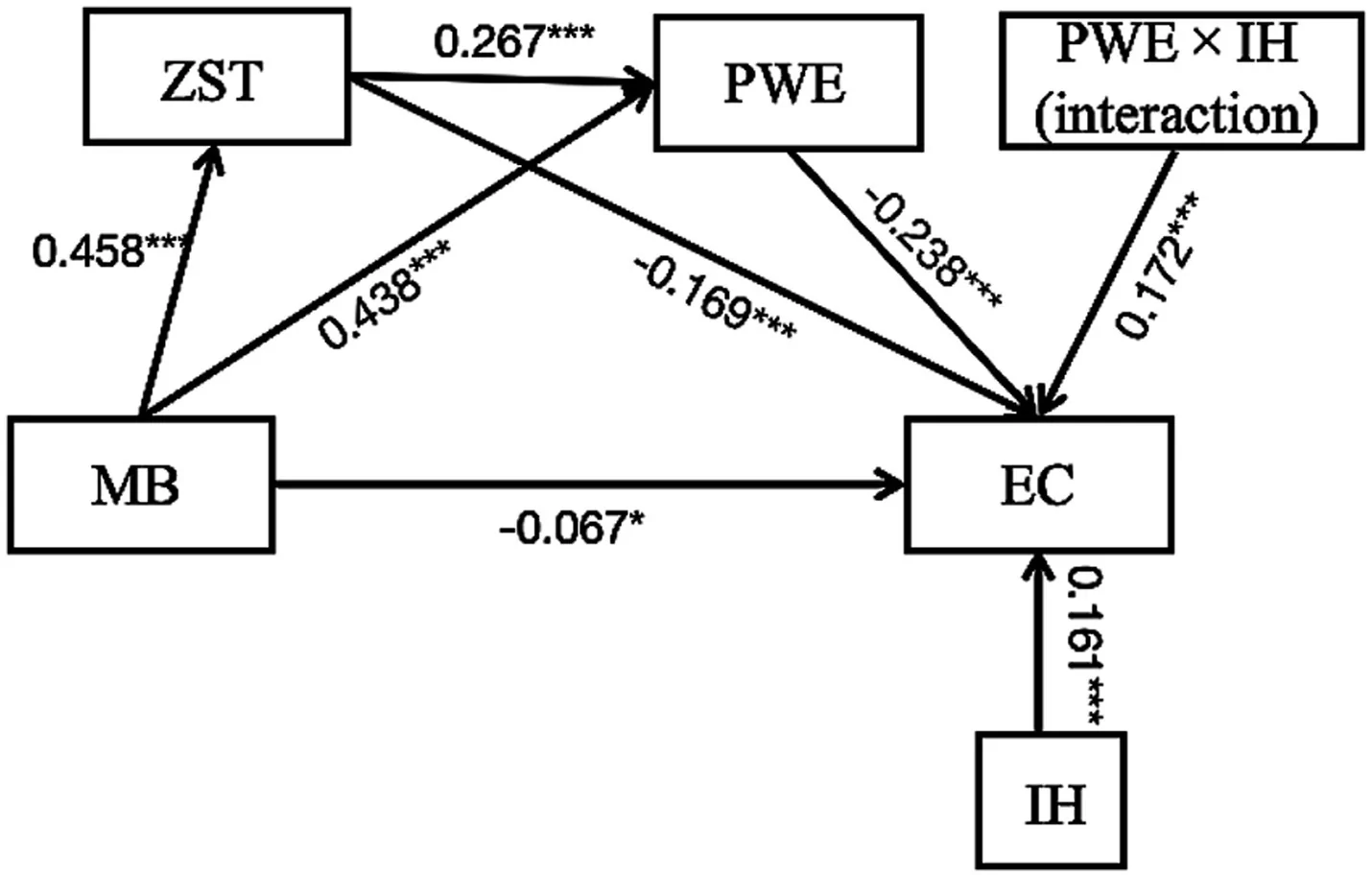
Moderated serial mediation model of Zero-Sum Thinking and Protestant Work Ethic in the relationship between Meritocracy Belief and Empathic Concern. MB, meritocracy belief; EC, empathic concern; ZST, zero-sum thinking; PWE, protestant work ethic; IH, intellectual humility. The interaction term represents the product of Protestant Work Ethic and Intellectual Humility (PWE × IH). Path coefficients are standardized coefficients (*β*). *p < 0.05, **p < 0.01, ***p < 0.001.

Structural path analysis indicated that the hypothesized associations were statistically significant (see Table 3 and Figure 1). The reported path coefficients are standardized coefficients (*β*). Meritocracy belief was positively associated with zero-sum thinking (*β* = 0.458, *p* < 0.001) and Protestant work ethic (*β* = 0.438, *p* < 0.001), while its direct association with empathic concern was statistically significant but relatively small in magnitude (*β* = −0.067, *p* = 0.034). Zero-sum thinking was positively associated with Protestant work ethic (*β* = 0.267, *p* < 0.001) and negatively associated with empathic concern (*β* = −0.169, *p* < 0.001). Protestant work ethic was negatively associated with empathic concern (*β* = −0.238, *p* < 0.001). The structural model explained 16.7% of the variance in empathic concern, 24.0% of the variance in zero-sum thinking, and 24.7% of the variance in Protestant work ethic.

**Table 3:** Path analysis.

Path Relationships	*β*	SE	*z*	*p*	95% CI
Direct paths					
MB → ZST	0.458	0.022	21.236	<0.001	[0.415, 0.500]
ZST → PWE	0.267	0.023	11.364	<0.001	[0.220, 0.313]
PWE → EC	−0.238	0.029	−8.245	<0.001	[−0.296, −0.182]
MB → PWE	0.438	0.022	19.732	<0.001	[0.394, 0.482]
ZST → EC	−0.169	0.028	−5.941	<0.001	[−0.228, −0.116]
MB → EC	−0.067	0.031	−2.127	0.034	[−0.127, −0.001]
Moderation effect paths					
IH → EC	0.161	0.024	6.632	<0.001	[0.110, 0.206]
PWE × IH → EC	0.172	0.023	7.389	<0.001	[0.121, 0.219]

Standardized coefficients (β) are reported. 95% CI represents the 95% confidence interval. MB, meritocracy belief; EC, empathic concern; ZST, zero-sum thinking; PWE, protestant work ethic; IH, intellectual humility.

Bootstrap analyses were conducted to estimate the total effect, total indirect effect, and specific indirect effects of meritocracy belief on empathic concern through the proposed mediating pathways. The total effect of meritocracy belief on empathic concern was −0.280, and the total indirect effect through the proposed mediators was −0.213. Specifically, meritocracy belief was indirectly associated with empathic concern through zero-sum thinking (indirect effect = −0.079) and through Protestant work ethic (indirect effect = −0.105). In addition, a significant sequential indirect association was observed through the pathway of zero-sum thinking → Protestant work ethic (indirect effect = −0.029). These findings indicate that zero-sum thinking and Protestant work ethic represent important indirect pathways linking meritocracy belief and empathic concern.

Moderation analysis showed that intellectual humility was positively associated with empathic concern (*β* = 0.158, *p* < 0.001). Moreover, the interaction between Protestant work ethic and intellectual humility (PWE × IH) was significantly associated with empathic concern (*β* = 0.170, *p* < 0.001), indicating that intellectual humility moderated the association between Protestant work ethic and empathic concern. Further simple slope analysis and conditional indirect effect analysis were conducted to examine the moderating pattern.

### 4.5 Testing of moderation and moderated serial mediation effects

**Figure 2:**
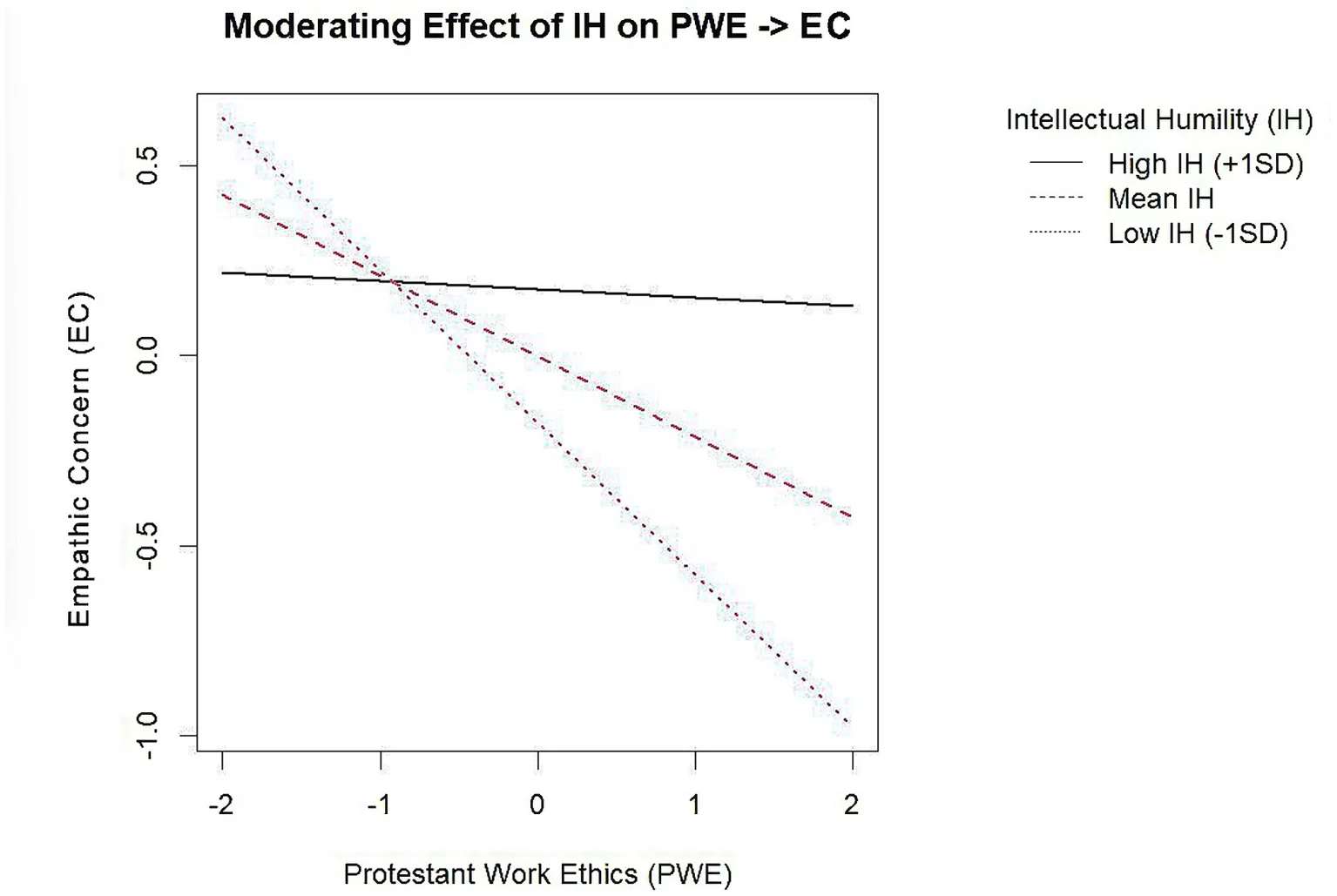
Simple slope plot.

To further examine the moderating role of intellectual humility in the association between Protestant work ethic and empathic concern, simple slope analyses were conducted. Intellectual humility was grouped into low (*M*-1SD), medium (*M*), and high (*M* + 1SD) levels to examine the association between Protestant work ethic and empathic concern at different levels of intellectual humility (see Table 4). The results showed that at a low level of intellectual humility (*M*-1SD), Protestant work ethic was significantly negatively associated with empathic concern (*β* = −0.401, *p* < 0.001). This negative association remained significant at a medium level of intellectual humility (*M*) (*β* = −0.212, *p* < 0.001), but became non-significant at a high level of intellectual humility (*M* + 1SD) (*β* = −0.022, *p* = 0.554). These findings indicate that intellectual humility moderated the association between Protestant work ethic and empathic concern, such that the negative association was weaker among individuals with higher levels of intellectual humility. Figure 2 illustrates this moderating pattern.

**Table 4:** Simple slope tests.

Level of IH	*β*	SE	*z*	*p*
Low (*M*-1SD)	−0.401	0.041	−9.686	<0.001
Medium (*M*)	−0.212	0.027	−7.862	<0.001
High (*M* + 1SD)	−0.022	0.038	−0.591	0.554

Standardized coefficients (β) are reported. IH, intellectual humility.

Following the moderation analysis, conditional indirect effects were further examined to determine whether the indirect association between meritocracy belief and empathic concern through Protestant work ethic varied across levels of intellectual humility. The Bootstrap results are presented in Table 5. The indirect association was significant at a low level of intellectual humility (*M* − 1SD) (*β* = −0.179, 95% CI [−0.218, −0.141]) and remained significant at a medium level (*M*) (*β* = −0.105, 95% CI [−0.132, −0.077]). However, this indirect association became non-significant at a high level of intellectual humility (*M* + 1SD) (*β* = −0.030, 95% CI [−0.063, 0.002]). The moderated mediation index was significant (Index = 0.074, 95% CI [0.052, 0.097]), suggesting that intellectual humility moderated the indirect association between meritocracy belief and empathic concern through Protestant work ethic.

**Table 5:** Moderated simple mediation effects.

Path	Level of IH	*β*	*p*	95% CI
MB → PWE → EC	Low (*M*-1SD)	−0.179	<0.001	[−0.218, −0.141]
	Medium (*M*)	−0.105	<0.001	[−0.132, −0.077]
	High (*M* + 1SD)	−0.030	0.07	[−0.063, 0.002]
Index	–	0.074	<0.001	[0.052, 0.097]

Standardized coefficients (β) are reported. 95% CI represents the 95% confidence interval. MB, meritocracy belief; EC, empathic concern; PWE, protestant work ethic; IH, intellectual humility.

Finally, the moderated serial mediation effect was examined to determine whether intellectual humility moderated the sequential indirect association from meritocracy belief to empathic concern through zero-sum thinking and Protestant work ethic. The results are presented in Table 6. The sequential indirect association was significant at a low level of intellectual humility (*M* − 1SD) (*β* = −0.050, 95% CI [−0.063, −0.037]) and remained significant at a medium level of intellectual humility (*M*) (*β* = −0.029, 95% CI [−0.038, −0.020]). However, the sequential indirect association became non-significant at a high level of intellectual humility (*M* + 1SD) (*β* = −0.009, 95% CI [−0.018, 0.001]). The moderated mediation index was significant (Index = 0.021, 95% CI [0.014, 0.028]), suggesting that intellectual humility moderated the sequential indirect association between meritocracy belief and empathic concern through zero-sum thinking and Protestant work ethic.

**Table 6:** Moderated serial mediation effects.

Path	Level of IH	*β*	*p*	95% CI
MB → ZST → PWE → EC	Low (*M*-1SD)	−0.050	<0.001	[−0.063, −0.037]
	Medium (*M*)	−0.029	<0.001	[−0.038, −0.020]
	High (*M* + 1SD)	−0.009	0.073	[−0.018, 0.001]
Index	–	0.021	<0.001	[0.014, 0.028]

Standardized coefficients (β) are reported. 95% CI represents the 95% confidence interval. MB, meritocracy belief; EC, empathic concern; ZST, zero-sum thinking; PWE, protestant work ethic; IH, intellectual humility.

## 5 Discussion

Based on Bandura’s Social Cognitive Theory (SCT; Bandura, 1986), this study examined the association between meritocracy belief and college students’ empathic concern, with a particular focus on the cognitive pathways and boundary conditions underlying this relationship. The findings supported the proposed model by showing that the association between meritocracy belief and empathic concern was primarily reflected through indirect pathways involving zero-sum thinking and Protestant work ethic. Although the direct association between meritocracy belief and empathic concern was statistically significant, its magnitude was relatively small. Furthermore, zero-sum thinking and Protestant work ethic emerged as independent and sequential cognitive pathways associated with this relationship, while intellectual humility moderated the association between Protestant work ethic and empathic concern. These findings extend existing research on meritocracy by highlighting its potential relevance to social–emotional outcomes and by revealing how achievement-related beliefs may be linked to interpersonal responses through cognitive processes.

### 5.1 Indirect cognitive pathways linking meritocracy belief and empathic concern

The present study’s core contribution lies in unpacking the cognitive mechanisms underlying the association between meritocracy belief and college students’ empathic concern. Statistically, the direct association between meritocracy belief and empathic concern reached significance, yet its magnitude was relatively small, indicating limited practical significance. In contrast, the indirect pathways accounted for most of the overall association between meritocracy belief and empathic concern. This pattern suggests that meritocracy belief is primarily associated with empathic concern through three cognitive pathways, including the independent mediating paths through zero-sum thinking and Protestant work ethic, as well as the sequential pathway from zero-sum thinking to Protestant work ethic. Accordingly, the following discussion focuses on these indirect cognitive pathways.

First, zero-sum thinking functions as an important cognitive pathway through which meritocracy beliefs are associated with empathic concern. Individuals who strongly endorse meritocracy beliefs may be more likely to interpret achievement opportunities through a competitive lens, perceiving success as involving limited and contested resources. This perspective may strengthen the perception that one person’s gain is linked to others’ loss, thereby reducing attention to others’ circumstances and weakening empathic concern. This interpretation aligns with previous studies suggesting that resource scarcity perceptions may suppress prosocial responses (Sun and Xiong, 2023; Wang et al., 2026). When individuals perceive resources as limited and substitutable, they may become more likely to evaluate interpersonal relationships through a competitive social perception, decreasing understanding and emotional input to other people’s conditions (Chun et al., 2023). Therefore, this pathway demonstrates that the association between meritocracy belief and empathic concern is partly explained by how students cognitively interpret peer competition.

Second, Protestant work ethic acts as a second independent mediating channel transmitting meritocracy belief’s negative association with empathic concern. Unlike meritocracy beliefs, which concern whether academic success is determined by individual merit, Protestant work ethic reflects the extent to which effort itself is viewed as a morally valued basis for evaluating outcomes. Individuals who strongly endorse meritocracy beliefs may therefore be more likely to further internalize effort as a meaningful explanation for differences in success and failure. Related studies have noted that individuals who emphasize performance and effort tend to attribute success and failure to personal effort and regard effort as morally significant (Amos et al., 2019; Celniker et al., 2023). Extending this perspective, the present study suggests that such effort-based evaluations may influence not only judgments of one’s own outcomes but also interpretations of others’ difficulties. According to attribution theory of responsibility, when others’ hardships are interpreted as internal and controllable, they are more likely to elicit blame and weaken empathic concern (Weiner, 1985; Christopher and Schlenker, 2005). Accordingly, meritocracy belief may be associated with students’ empathic responses indirectly through an effort-centered evaluative framework, which may narrow their attention to situational barriers that may contribute to others’ difficulties.

Further analysis revealed a sequential cognitive pathway linking meritocracy beliefs and empathic concern through zero-sum thinking and Protestant work ethic. This finding suggests that meritocracy beliefs may relate to empathic concern through a series of connected beliefs about competition and personal responsibility. Specifically, individuals who strongly endorse meritocracy beliefs may be more likely to perceive achievement opportunities as competitive, which may strengthen zero-sum thinking. Such competitive perceptions may further increase the tendency to explain differences in outcomes through personal effort and responsibility. In this process, individuals may rely more on effort-based judgments when interpreting others’ situations, thereby reducing attention to contextual factors underlying others’ difficulties. This sequential pathway integrates competitive perceptions and responsibility attribution, providing further insight into how meritocracy beliefs may be associated with empathic concern.

Overall, the findings support the SCT perspective that beliefs are associated with emotional responses through cognitive processes (Bandura, 1986, 2001). Although the direct association between meritocracy beliefs and empathic concern was relatively weak, the indirect pathways through zero-sum thinking and Protestant work ethic highlight the importance of cognitive interpretations in understanding this relationship. Previous research has mainly examined meritocracy beliefs in relation to achievement motivation and judgments of fairness, whereas the present study extends this perspective by showing that meritocracy beliefs may also be associated with interpersonal responses through the ways individuals interpret competition, effort and responsibility. These findings suggest that meritocracy beliefs may have different implications across domains, as they may support individuals’ pursuit of achievement while also being associated with reduced empathic concern when used as a framework for evaluating others’ circumstances.

### 5.2 The moderating effect of intellectual humility and the formation of moderated mediation

This study further identified intellectual humility as an important boundary condition in the relationship between Protestant work ethic and empathic concern. The findings suggest that the negative association between effort-centered beliefs and empathic concern may depend on how individuals process and apply these beliefs. From the perspective of SCT, individual characteristics can influence how cognitive beliefs are translated into emotional and behavioral responses (Bandura, 2001). As a personality characteristic involving recognition of one’s cognitive limitations and openness to different viewpoints (Leary et al., 2017), intellectual humility may reduce the tendency to rely on a single perspective when interpreting social situations. Previous research has also suggested that intellectual humility is associated with greater openness and reduced reliance on rigid judgments (Brandt et al., 2014; Porter et al., 2022). Therefore, even when individuals value effort and personal responsibility, those with higher intellectual humility may be more likely to consider situational factors and alternative explanations when evaluating others’ difficulties, thereby maintaining empathic concern.

Beyond its moderating role in the association between Protestant work ethic and empathic concern, intellectual humility also moderated the sequential pathway linking meritocracy beliefs to empathic concern through zero-sum thinking and Protestant work ethic. This finding suggests that intellectual humility may influence not only a specific cognitive link but also the extent to which competitive beliefs are translated into emotional responses. Importantly, the buffering role of intellectual humility does not appear to result from reducing individuals’ endorsement of meritocracy beliefs, zero-sum thinking, or Protestant work ethic. Instead, individuals with higher intellectual humility may maintain these beliefs while recognizing the limitations of their own judgments and applying them in a more flexible and reflective manner (Haggard et al., 2018). Although they recognize the importance of effort and personal responsibility, they may also consider situational factors and alternative explanations when evaluating others’ difficulties (Krumrei-Mancuso, 2017). Consistent with this interpretation, intellectual humility was positively correlated with meritocracy belief, zero-sum thinking, and Protestant work ethic, suggesting that intellectual humility does not weaken competitive beliefs themselves but changes how these beliefs are interpreted and applied. This finding extends SCT by highlighting the role of individual characteristics in shaping the process through which cognitive beliefs are associated with emotional responses.

## 6 Conclusion and implications

### 6.1 Conclusion

Based on Bandura’s Social Cognitive Theory, this cross-sectional correlational study examined how meritocracy belief was associated with empathic concern among Chinese college students, focusing on the sequential mediating roles of zero-sum thinking and Protestant work ethic and the moderating role of intellectual humility. The findings showed that the direct association between meritocracy belief and empathic concern was statistically significant but weak in magnitude. More importantly, meritocracy belief was primarily linked to empathic concern through cognitive pathways involving zero-sum thinking and Protestant work ethic, with intellectual humility serving as an important boundary condition. Overall, this study highlights the complex role of meritocracy beliefs in students’ interpersonal responses, suggesting that beliefs supporting achievement striving may also shape how individuals understand and evaluate others’ circumstances in competitive educational contexts.

### 6.2 Theoretical implications

This research extends the application of Social Cognitive Theory by explaining how internalized value beliefs are associated with interpersonal affective responses in educational contexts. First, it distinguishes individual meritocracy belief from institutional meritocratic environments and clarifies how personal value beliefs are linked to empathic concern through cognitive processes. Second, it integrates zero-sum thinking and Protestant work ethic into a sequential framework, providing a more comprehensive explanation of how competitive perceptions and effort-based evaluations jointly shape interpersonal responses. Third, it highlights intellectual humility as an important boundary condition, suggesting that individual characteristics influence how meritocratic and effort-related beliefs are interpreted and applied. The findings also provide insights into the operation of these processes within a Chinese higher education context.

### 6.3 Practical implications

This study suggests that meritocracy beliefs are primarily associated with college students’ empathic concern through cognitive processes involving zero-sum thinking and Protestant work ethic, highlighting the need to balance achievement pursuit with interpersonal development in educational contexts. First, educational practices should pay attention to the cognitive consequences of highly competitive evaluation systems. Educators may reduce excessive emphasis on social comparison and single performance indicators to prevent students from viewing achievement opportunities through a zero-sum lens and encourage recognition of diverse pathways to success. Second, students should be guided to adopt more balanced interpretations of success and failure rather than relying solely on personal effort when evaluating outcomes, thereby reducing responsibility-focused judgments of others. Third, given the protective role of intellectual humility, learning activities that encourage openness to different viewpoints, and respect for diverse experiences may help students develop more flexible ways of understanding others in competitive contexts.

### 6.4 Research limitations and future research directions

Several limitations of the present study should be acknowledged. First, the cross-sectional design limits the ability to determine the temporal and causal relationships among meritocracy belief, cognitive orientations, and empathic concern. Future research could adopt longitudinal or experimental designs to further examine these relationships. Second, although the proposed sequential pathway was theoretically grounded, alternative cognitive sequences or explanatory mechanisms may also account for the association between meritocracy belief and empathic concern. Future research could compare alternative models or use longitudinal designs to further clarify the ordering of these processes. Third, the cultural meaning of Protestant work ethic requires further examination. In the Chinese context, effort, diligence, and academic striving may also reflect broader cultural values rather than Protestant work ethic alone. Future research should explore how effort-related beliefs operate across different cultural contexts and whether their psychological meanings vary across populations. Finally, the sample was limited to Chinese college students recruited through an online survey platform, which may restrict the generalizability of the findings. Future research should examine the proposed model across diverse populations and cultural contexts while further testing measurement equivalence across groups.

## Data Availability

The original contributions presented in the study are included in the article/supplementary material, further inquiries can be directed to the corresponding author.
